# Luteolin Inhibits Angiotensin II-Stimulated VSMC Proliferation and Migration through Downregulation of Akt Phosphorylation

**DOI:** 10.1155/2015/931782

**Published:** 2015-08-10

**Authors:** Tongda Xu, Hong Zhu, Dongye Li, Yasong Lang, Lijuan Cao, Yang Liu, Wanling Wu, Dan Chen

**Affiliations:** ^1^Institute of Cardiovascular Disease Research, Xuzhou Medical College, 84 West Huaihai Road, Xuzhou, Jiangsu 221002, China; ^2^Department of Cardiology, Affiliated Hospital of Xuzhou Medical College, 99 West Huaihai Road, Xuzhou, Jiangsu 221002, China

## Abstract

Luteolin is a naturally occurring flavonoid found in many plants that possesses cardioprotective properties. The purpose of this study was to elucidate the effect of luteolin on vascular smooth muscle cells (VSMCs) proliferation and migration induced by Angiotensin II (Ang II) and to investigate the mechanism(s) of action of this compound. Rat VSMCs were cultured *in vitro*, and the proliferation and migration of these cells following Ang II stimulation were monitored. Different doses of luteolin were added to VSMC cultures, and the proliferation and migration rate were observed by MTT and Transwell chamber assays, respectively. In addition, the expressions of p-Akt (308), p-Akt (473), and proliferative cell nuclear antigen (PCNA) in VSMCs were monitored by Western blotting. This study demonstrated that luteolin has an inhibitory effect on Ang II-induced VSMC proliferation and migration. Further, the levels of p-Akt (308), p-Akt (473), and PCNA were reduced in VSMCs treated with both Ang II and luteolin compared to VSMCs treated with only Ang II. These findings strongly suggest that luteolin inhibits Ang II-stimulated proliferation and migration of VSMCs, which is partially due to downregulation of the Akt signaling pathway.

## 1. Introduction

Atherosclerosis (AS) is a chronic inflammatory disease of arteries [1], and the proliferation and migration of vascular smooth muscle cells (VSMCs) play important roles in the pathogenesis and development of AS. Angiotensin II (Ang II) is well-characterized and important factor in the renin-angiotensin-aldosterone system. In addition to maintaining the volume of circulating blood and regulating blood pressure, Ang II also is a factor in chronic inflammation and mediates various responses, such as cell activation, proliferation, migration, and apoptosis [2, 3].

Abnormal VSMCs proliferation and migration have been implicated in a number of cardiovascular pathologies, including the pathogenesis of hypertension, the development of restenosis after percutaneous coronary intervention (PCI), and the progression of AS [4, 5]. In the context of AS [6], or in response to arterial endothelial denudation [7], the normally quiescent VSMCs of the tunica media of the artery proliferate and migrate into the subendothelial intima, thereby driving AS progression.

Akt is a serine/threonine protein kinase and the Akt signaling pathway is involved in the regulation of multiple biological processes, including cell survival, proliferation, migration, and glycogen metabolism [8]. Akt is essential for VSMCs proliferation and migration, and the ablation of Akt leads to a severe lesion in AS and occlusive artery disease [9]. This factor is activated by phosphorylation on two critical residues, namely, threonine 308 (Thr308) and serine 473 (Ser473), and is regulated by upstream second messengers as well as other enzymes [10]. Akt is recruited to the cell membrane, where it is activated by 3-phosphoinositide-dependent kinase 1 (PDK1). Inhibition of Ang II-stimulated VSMCs migration and proliferation via therapeutic intervention is an important step towards attenuating vascular diseases and preventing restenosis after PCI [11].

Many studies have reported that dietary flavonoids provide protection against VSMCs disorders [12–15]. Luteolin is a common dietary flavonoid that has been reported to possess a wide range of pharmacological effects, including anti-inflammatory and antiproliferative activities. Studies of the antitumor effects of luteolin and its related mechanisms have been reported frequently in recent years [16]. However, an examination of the effect of luteolin on VSMCs proliferation and migration has not yet been reported.

In the present study, we carried out a preliminary study on the effects of luteolin on Ang II-induced VSMCs proliferation and migration. To further evaluate the effects of luteolin on the regulation of Ang II-induced VSMCs at a nuclear level, PCNA expression is examined. Furthermore, we also explored the hypothesis of the antiproliferative and antimigration effects of luteolin by inhibition of the Akt/PCNA signaling pathway, one of the many interrelated underlying mechanisms.

## 2. Methods 

### 2.1. Main Reagents and Antibodies

Luteolin was obtained from Sigma (St. Louis, USA), dissolved in dehydrated alcohol, and stored at 4°C until further use. Ang II was purchased from Beijing Union Pharmaceutical Factory (Beijing, China), dissolved in sterile deionized water, and stored at 4°C until later use. Fetal bovine serum (FBS) was acquired from Gibco/Invitrogen (Grand Island, NY, USA). The assay reagent 3-(4,5-dimethylthiazol-2-yl)-2,5-diphenyltetrazolium bromide (MTT) was purchased from Sigma (St. Louis, USA). Transwell chambers were manufactured by Millipore (Germany). An anti-PCNA polyclonal antibody and phosphospecific polyclonal antibodies against p-Akt (308) and p-Akt (473) were purchased from Cell Signaling Technology (Danvers, USA). The anti-Akt polyclonal and anti-PCNA polyclonal antibody was obtained from Zhongshan Golden Bridge.

### 2.2. Cell Culture and Treatment Schedule

Thoracic aortas were quickly excised from sacrificed Sprague-Dawley rats (8–10 weeks old, male or female, weighed 200 ± 20 g, provided by the Institute of Laboratory Animal of Xuzhou Medical College). Rat aortic VSMCs were harvested using the explants culture technique, incubated at 37°C in a 5% CO_2_ atmosphere, and maintained in Dulbecco's modified Eagle's medium (DMEM; Hyclone) containing FBS (20% FBS for primary culture and 10% FBS for subculture), penicillin (100 IU/mL), and streptomycin (100 *μ*g/mL). The purity of VSMCs cultures was confirmed with an antibody against smooth muscle *α*-actin (DAKO Corp.; Glostrup, Denmark). For all experiments, only cell passages 3–5 were used. Before each experiment, cells were serum starved for 24 h in DMEM containing 5.5 mM glucose and 1% antibiotics. The experimental VSMCs were given 12.5 *μ*M (low-dose group), 25 *μ*M (middle-dose group), and 50 *μ*M (high-dose group) of luteolin. VSMCs were divided into groups and treated with Ang II for different lengths of times, with luteolin for different periods of time, or with luteolin at different concentrations.

### 2.3. Cell Viability/Proliferation Assay

MTT was used to measure the viability and proliferation of VSMCs. VSMCs at logarithmic growth phase were digested, centrifuged, and inoculated into 96-well flat-bottomed plates. Each well contained 100 *μ*L of VSMCs suspension, with 1 × 10^4^ cells per well. After being cultured for 24 h, VSMCs were treated with serum-free media for about 24 h to achieve synchronization at G_0_/G_1_ phase. After pretreatment with different concentrations of luteolin for 12 h, VSMCs were stimulated with Ang II. MTT solution (5 mg/mL) was added to each well, after incubation for 4 h at 37°C. The cell culture medium was removed and 100 *μ*L of DMSO was added to each well. Absorbance at 570 nm was measured for each well with an ELx800 Universal Microplate Reader (BIO-TEK, INC USA). The proliferation of the control VSMCs was considered to be 100%; results were expressed as relative proliferation rates.

### 2.4. Cell Migration Assay

The migration of the cultured cells was examined by Transwell chamber assay. The VSMCs were cultured in the upper chamber while only 10% FBS was placed in the lower chamber without cells. The cells were incubated at 37°C in a 5% CO_2_ atmosphere for 8 h. Depending on the experimental group to which they were assigned, VSMCs were cultured with Ang II (1 *μ*M) and different concentrations of luteolin for 12 h. The VSMCs migrated through the micropores and stayed on the bottom side of the Transwell chamber. The upper chamber was removed out from the 24 pore plates and the residual cells on the inner side of the Transwell filters were wiped with cotton swab. The Transwell filters were flushed with 0.01 M PBS, fixed with 4% paraformaldehyde, and washed for 2 min with distilled water. After HE staining, 5 visual fields were chosen at random from each of the Transwell filters, and the average number of cells that migrated through the Transwell filters was counted under the microscope.

### 2.5. Western Blotting Analysis

The cell culture medium was poured off and the culture plates were washed twice with PBS at 4°C. Cells were lysed with lysis buffer on ice for 30 min. During this procedure the culture plate was agitated from time to time to ensure that the lysis buffer fully covered the cells. The cell lysates were centrifuged at 12,000 g for 15 min at 4°C. Equal amounts of soluble protein were separated by electrophoresis on a 12% SDS-PAGE gel and proteins were then electrotransferred to a polyvinylidene fluoride membrane. The membranes were immunoblotted with the appropriate primary and secondary antibodies. The membranes were photographed with 5-bromo-4-chloro-3-indolyl phosphate/nitroblue-tetrazolium (BCIP/NBT) coloration and grey scale values were determined with the Image J 3.0 system (National Institutes of Health; MD, USA).

### 2.6. Statistical Analysis

All data were expressed as mean ± SEM. Statistical analysis was performed with GraphPad Prism 4.0. The statistical significance of differences was determined using ANOVA followed by Bonferroni correction for post hoc analysis. Differences were considered statistically significant when *P* < 0.05.

## 3. Results

### 3.1. Luteolin Inhibits Ang II-Induced VSMCs Proliferation

**Figure 1:**
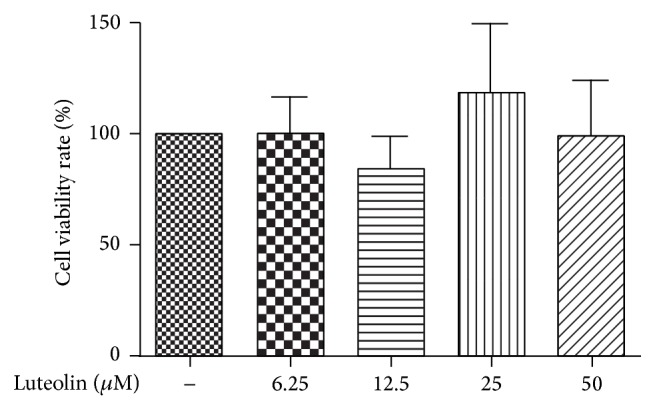
Investigating the cytotoxicity of luteolin in VSMCs. VSMCs were incubated with varying concentrations of luteolin (6.25, 12.5, 25, and 50 *μ*M) for 12 h, MTT reagent was added, and the OD value of each group was measured. The cell viability of the control group was taken as 100%.

**Figure 2:**
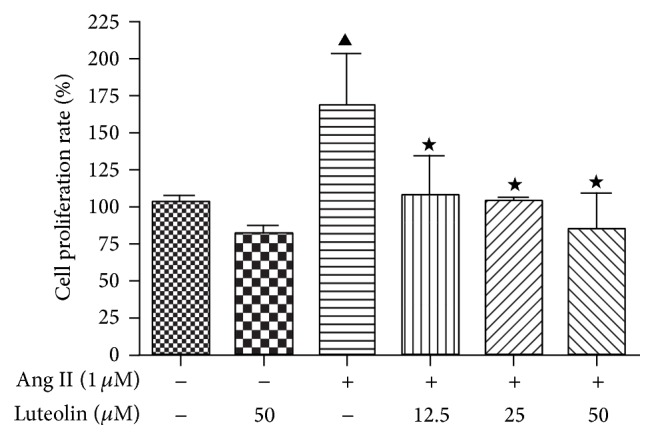
Inhibitive effect of luteolin on VSMCs proliferation induced by Ang II. VSMCs were pretreated with the indicated concentrations of luteolin for 12 h prior to being stimulated with 1 *μ*M Ang II. MTT assays were performed to measure cell proliferation, and the cell proliferation rate of the control group was taken as 100%. Data are shown as the mean ± SEM of 3 independent experiments. ^▲^ *P* < 0.05 compared with the control group; ^★^ *P* < 0.05 compared with the Ang II group.

To test for cytotoxic effects of luteolin on VSMCs viability or cell proliferation, VSMCs were pretreated with luteolin or vehicle for 12 h. The MTT assay was then used to measure the viability and proliferation of VSMCs. As shown in Figure 1, cytotoxicity was not observed at luteolin concentrations up to 50 *μ*M (*P* > 0.05). As seen in Figure 2, VSMC treatment with 1 *μ*M Ang II significantly increases proliferation compared to the control group (*P* < 0.05).

### 3.2. Luteolin Suppresses Ang II-Stimulated VSMC Migration

**Figure 3:**
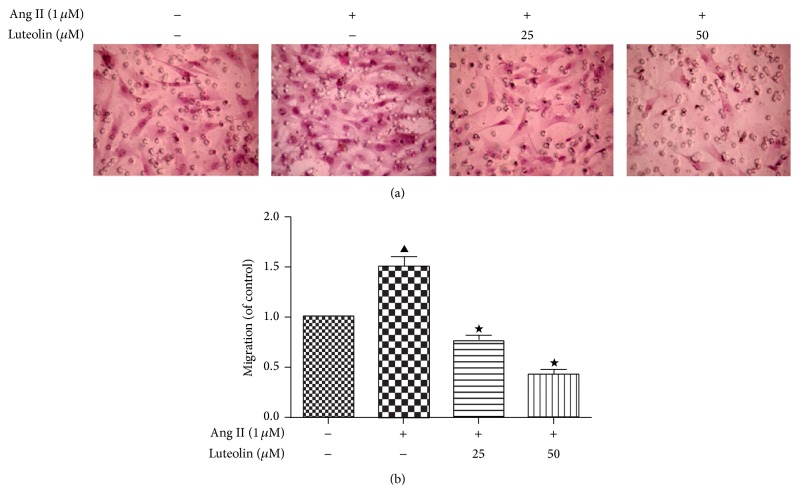
Inhibitory effect of luteolin on Ang II-induced VSMC migration. After being incubated with the indicated concentrations of luteolin, VSMCs were stimulated with 1 *μ*M Ang II. The VSMC migration rate was determined by Transwell chamber assay. Bright-field images of randomly selected squares per group (×100). The cell migration rate of the control group was taken as 1. ^▲^ *P* < 0.05 compared with the control group; ^★^ *P* < 0.05 compared with the Ang II group.

The migration of VSMCs following Ang II stimulation was examined by Transwell chamber assay. As shown in Figure 3, 1 *μ*M Ang II clearly promoted the migration of VSMCs from the upper chamber to the lower chamber as compared to the control group (*P* < 0.05). Pretreatment with luteolin for 12 h significantly suppressed the migration stimulated by Ang II in a dose-dependent manner (*P* < 0.05). Thus, as the concentration of luteolin increased, the number of cells migrating decreased.

### 3.3. Impact of Luteolin on Ang II-Induced Activation of Akt

**Figure 4:**
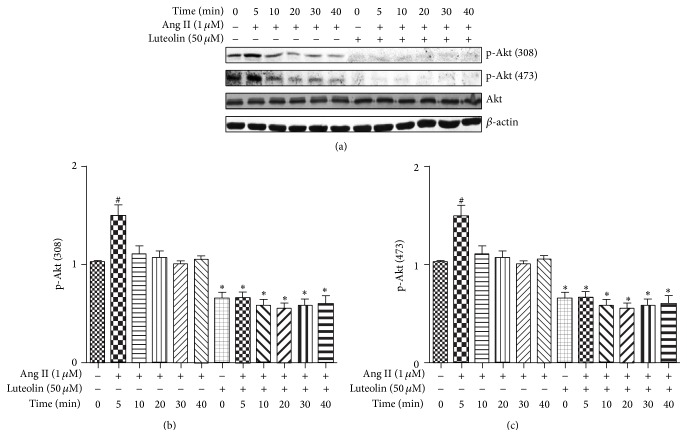
Effect of luteolin on different lengths of time on Ang II-induced activation of Akt. Following pretreatment with 50 *μ*M luteolin for 12 h, VSMCs were stimulated with Ang II (1 *μ*M) for the indicated times, and luteolin significantly attenuates Akt phosphorylation induced by Ang II. The cells were lysed and analyzed with antibodies against p-Akt (308), p-Akt (473), and Akt. ^#^ *P* < 0.05 compared with the control group; ^*∗*^ *P* < 0.05 compared with the Ang II group.

To examine whether luteolin inhibited Ang II-induced VSMCs proliferation or migration by affecting Akt activation, VSMCs were pretreated by luteolin for 12 h and then stimulated with 1 *μ*M Ang II for different lengths of time. The stimulatory effect of Ang II on the Akt pathway of VSMCs was apparent after only 5 min, and the effect lasted up to 40 min (see Figure 4). After 5 min of Ang II activation, p-Akt (308) and p-Akt (473) reached peak levels of activation (*P* < 0.05); phosphorylation then went back down to background levels. Our results demonstrated that Akt phosphorylation is increased by Ang II stimulation and is abolished by pretreatment with luteolin (*P* < 0.05).

**Figure 5:**
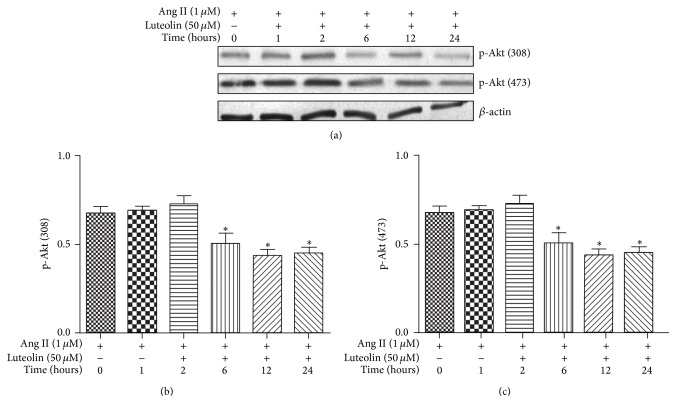
Effect of luteolin for different lengths of times on Ang II-induced activation of Akt. Following pretreatment with luteolin for different lengths of times, VSMCs were incubated with 1 *μ*M Ang II for 12 h. The cells were lysed and analyzed with the antibodies against p-Akt (308), p-Akt (473), and Akt. Equal protein loading was confirmed by comparison of *β*-actin levels. ^*∗*^ *P* < 0.05 compared with the Ang II group.

**Figure 6:**
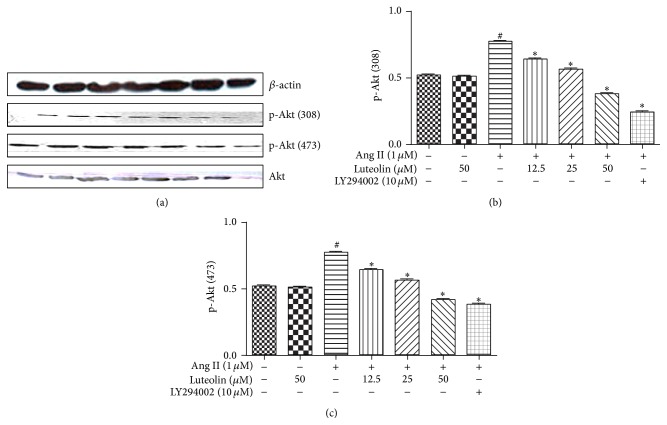
Effect of different concentrations of luteolin on Ang II-induced activation of Akt. After incubation with the indicated concentrations of luteolin, VSMCs were stimulated with 1 *μ*M Ang II. The cells were lysed and analyzed using the antibodies versus p-Akt (308), p-Akt (473), and Akt. Equal protein loading was confirmed by comparison of *β*-actin levels. ^#^ *P* < 0.05 compared with the control group; ^*∗*^ *P* < 0.05 compared with the Ang II group.

VSMCs were pretreated with various concentrations of luteolin for different lengths of time before stimulating for 12 h with 1 *μ*M Ang II. As shown in Figure 5, pretreatment with luteolin for 6 h could significantly suppress the Ang II-stimulated phosphorylation of p-Akt (308) and p-Akt (473) (*P* < 0.05). As shown in Figure 6, luteolin can significantly suppress the phosphorylation of p-Akt (308) and p-Akt (473) stimulated by Ang II (*P* < 0.05). LY294002 (Akt inhibitor; 10 *μ*M) was used as a positive control in these experiments.

### 3.4. Luteolin Inhibits Ang II-Stimulated PCNA Expression in VSMCs

**Figure 7:**
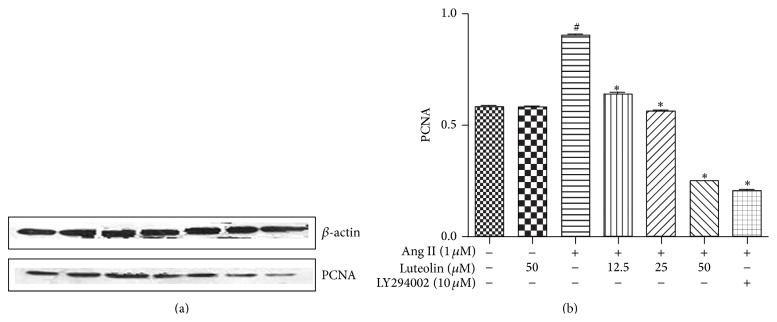
Inhibitory effects of luteolin on PCNA expression in Ang II-stimulated VSMCs. The cells were lysed and analyzed using the antibodies versus PCNA. Equal protein loading was confirmed by comparison of *β*-actin levels. ^#^ *P* < 0.05 compared with the control group; ^*∗*^ *P* < 0.05 compared with the Ang II group.

To estimate the inhibitory effect of luteolin on VSMCs proliferation at the nuclear level, the expression of PCNA was detected. As shown in Figure 7, Ang II-induced PCNA expression was significantly inhibited by luteolin in a concentration-dependent manner. The percentage inhibition observed with 12.5, 25, and 50 *μ*M luteolin was 29.16%, 38.58%, and 66.86%, respectively.

## 4. Discussion

Due to various changes in social and living conditions, the morbidity and mortality of cardiovascular diseases have increased, with AS being one of the leading causes. Consequently, elucidation of the molecular and cellular mechanisms that drive the development and progression of AS is necessary for formulating strategies to treat and prevent AS. However, due to its complicated causes and pathologies, there are relatively few safe and effective drugs for treating AS. Therefore, the search for natural, inexpensive, and effective anti-AS drugs with little or no adverse reactions has become an intense area of research.

There are many cells that play a role in the formation of AS, and the activities of VSMCs are particularly important. AS and arterial injury-induced neointimal hyperplasia involve medial VSMCs proliferation and migration into the arterial intima. The media of vessels chiefly consist of VSMCs, which have the ability to shrink and differentiate in normal culture conditions. However, VSMCs are prone to hypertrophy and lose their differentiation capacity due to the action of many pathological factors. VSMCs can synthesize high levels of extracellular matrix and inflammatory factors, proliferate, and migrate to the intima in excess. A high restenosis rate and poor long-term therapeutic outcomes following PCI have become the important factors driving the development of PCI procedures.

Many published studies support anti-AS and cardioprotective roles for dietary flavonoids that are ubiquitous in fruits and vegetables [17]. The results of domestic and international studies indicate that luteolin has anticancer, antioxidation, and anti-inflammatory roles, removes free radicals, and protects against cardiomyocyte ischemia/reperfusion (I/R) injury [18–20]. VSMCs proliferation and migration, as well as endothelial cell apoptosis, are important processes that participate in the pathogenesis of atherosclerosis. Recent reports demonstrate that luteolin inhibits lysophosphatidylcholine- (LPC-) and Ang II-induced apoptosis through downregulation of PI3K/Akt signaling pathway in endothelial cells [21, 22]. These reports can suggest that luteolin may act against AS. Meanwhile, in a similar manner, our presented results reveal that luteolin suppresses H_2_O_2_-induced proliferation and migration in VSMCs by attenuating the phosphorylation of Akt, published in journal of Pharmacy and Pharmacology (JPP) by Lang et al. [23]. In the paper of JPP, as an oxidative stimulant, H_2_O_2_ is one of the most important molecules of signal transduction in ROS [24], which can promote the proliferation and migration of VSMCs by the mechanism of oxidative stress; thereby luteolin can play an important role in inhibiting the proliferation and migration of VSMCs by attenuating the expression of PDK1 mediated p-Akt and p-Src from the molecular mechanism of oxidative stress.

Some documents have shown that the mechanisms for apoptosis, inflammatory response, oxidative stress, and so forth maybe be involved in proliferation and migration of VSMCs. Ang II can be more extensively involved in the production for inflammatory factor, oxygen free radical, and apoptosis related protein than that of H_2_O_2_-induced oxidative stress [25, 26]. So in our recent study, to further clarify the mechanism of inhibiting proliferation and migration of VSMCs by luteolin pretreatment, Ang II is used to induce proliferation and migration of VSMCs.

VSMC proliferation and migration are crucial events underlying the complications of vascular injury. The purpose of the present study was to determine which effect was played on VSMC proliferation and migration by luteolin pretreatment. The present study demonstrated that VSMC proliferation and migration can be significantly induced by 1 *μ*M Ang II, while pretreatment with 50 *μ*M luteolin significantly suppressed this proliferation and migration without any cytotoxic effects.

Acting as a proinflammatory factor, Ang II plays a crucial role in the pathogenesis of cardiovascular diseases [27, 28]. Ang II can stimulate the proliferation and migration of VSMCs, processes that are critically involved in AS, postangioplasty restenosis, and other inflammatory vascular diseases. Ang II can also activate a series of protein kinases such as Akt, thereby activating downstream signaling cascades driving the pathogenesis of AS.

The Akt pathway is crucial for mediating changes in gene expression that regulate cell survival, proliferation, migration, and growth. The phosphorylation of both its activation sites, Thr308 and Ser473, is essential for the full activation of Akt. It has been suggested that different signaling pathways regulate the proliferation and migration response to Ang II, and our findings indicate that the effects of luteolin on VSMCs may be mediated by a coordinated inhibition of the Akt signaling pathway.

To further evaluate the effects of luteolin on the regulation of VSMCs at a nuclear level, we examined PCNA expression. PCNA is known to be synthesized during the early G_1_ and S phases of the cell cycle and to behave as a marker of cell proliferation in both normal and disease states, usually assayed in tumor cell not for VSMCs [29, 30]. In this study, we demonstrated that luteolin suppresses PCNA expression, suggesting that luteolin can directly inhibit VSMC growth at the nuclear level; meanwhile, the similar research has not been reported.

## 5. Conclusion

In conclusion, with the ultimate goal of preventing AS and restenosis following angioplasty, the identification and characterization of effective compounds that can inhibit VSMCs proliferation and migration have become one hot spot of investigation. The results obtained from this study suggest that luteolin can inhibit Ang II-stimulated VSMC proliferation and migration. The effect of luteolin is partially associated with inhibition of the PI3K/Akt pathway. Additionally, this study shows that luteolin inhibits the Ang II-induced expression of PCNA.

Further studies centered on the detailed molecular mechanisms of luteolin are now in progress. We hope that our study lays a solid foundation for the clinical application of luteolin for treatment of AS. As the proliferation of VSMCs is an important determinant of atherosclerotic plaque development and stability, luteolin may be a valuable therapeutic compound for the treatment of cardiovascular diseases in the near future.
